# Ubiquitous Overexpression of Chromatin Remodeling Factor SRG3 Exacerbates Atopic Dermatitis in NC/Nga Mice by Enhancing Th2 Immune Responses

**DOI:** 10.3390/ijms22041553

**Published:** 2021-02-04

**Authors:** Sung Won Lee, Hyun Jung Park, Jungmin Jeon, Yun Hoo Park, Tae-Cheol Kim, Sung Ho Jeon, Rho Hyun Seong, Luc Van Kaer, Seokmann Hong

**Affiliations:** 1 Department of Integrative Bioscience and Biotechnology, Institute of Anticancer Medicine Development, Sejong University, Seoul 05006, Korea; insiderjjang@gmail.com (S.W.L.); 0402parkhj@gmail.com (H.J.P.); jjm4165@gmail.com (J.J.); dbsgn703@gmail.com (Y.H.P.); mitalus1@gmail.com (T.-C.K.); 2 Department of Life Science and Multidisciplinary Genome Institute, Hallym University, Chuncheon 24252, Korea; sjeon@hallym.ac.kr; 3 School of Biological Sciences, Institute of Molecular Biology and Genetics, Seoul National University, Seoul 08826, Korea; rhseong@snu.ac.kr; 4 Department of Pathology, Microbiology and Immunology, Vanderbilt University School of Medicine, Nashville, TN 37232, USA; luc.van.kaer@vumc.org

**Keywords:** SWI3-related gene (SRG3), atopic dermatitis, Th2 cells, Treg cells, NC/Nga, WT

## Abstract

The SWItch (SWI)3-related gene (SRG3) product, a SWI/Sucrose Non-Fermenting (SNF) chromatin remodeling subunit, plays a critical role in regulating immune responses. We have previously shown that ubiquitous SRG3 overexpression attenuates the progression of Th1/Th17-mediated experimental autoimmune encephalomyelitis. However, it is unclear whether SRG3 overexpression can affect the pathogenesis of inflammatory skin diseases such as atopic dermatitis (AD), a Th2-type immune disorder. Thus, to elucidate the effects of SRG3 overexpression in AD development, we bred NC/Nga (NC) mice with transgenic mice where SRG3 expression is driven by the β-actin promoter (SRG3^β-actin^ mice). We found that SRG3^β-actin^ NC mice exhibit increased AD development (e.g., a higher clinical score, immunoglobulin E (IgE) hyperproduction, and an increased number of infiltrated mast cells and basophils in skin lesions) compared with wild-type NC mice. Moreover, the severity of AD pathogenesis in SRG3^β-actin^ NC mice correlated with expansion of interleukin 4 (IL4)-producing basophils and mast cells, and M2 macrophages. Furthermore, this accelerated AD development is strongly associated with Treg cell suppression. Collectively, our results have identified that modulation of SRG3 function can be applied as one of the options to control AD pathogenesis.

## 1. Introduction

Atopic dermatitis (AD) is a chronic inflammatory skin disease characterized by xerosis, pruritus, and erythematous lesions [1]. Most patients with AD exhibit disease parameters such as increased total immunoglobulin E (IgE) levels, eczema lesion areas, and Th2 polarity. In the pathogenesis of AD, the interplay between innate immune cells (mast cells, eosinophils, basophils, macrophages, and dendritic cells) and adaptive immune cells (B and T cells) plays an essential role in affecting disease outcome [2,3,4]. NC/Nga (NC) mice are a spontaneous model of AD and exhibit AD-like skin inflammation as well as increased serum IgE and Th2 cells when these animals are housed under conventional conditions [5,6,7].

Foxp3 (forkhead box P3), also known as scurfin, is a critically important transcription factor for controlling regulatory T (Treg) cell development and function. Foxp3^+^ Treg cells play pivotal roles in maintaining immunologic self-tolerance via modulation of both allergic and autoimmune responses [8,9,10,11]. It has been reported that Foxp3^+^ Treg cell infiltration is impaired in the skin lesions of patients with AD [12]. Further, Verhagen et al. showed that Treg cell depletion significantly exacerbates AD-like symptoms in a murine model [13].

SWItch/Sucrose Non-Fermentable (SWI/SNF) is an ATP-dependent chromatin remodeling complex, which regulates conformational changes of nucleosomes. SWI3-related gene (SRG3) is a core component of various SWI/SNF complexes [14]. SRG3 acts as a regulator of the development and function of T cells [15] and B cells [16]. We have shown that ubiquitous, beta-actin promoter-driven SRG3 (SRG3^β-actin^) overexpression in transgenic mice protects against experimental autoimmune encephalomyelitis (EAE) [17]. Moreover, these anti-inflammatory effects of SRG3 overexpression were associated with expansion and increased IL4 production of basophils and mast cells [17].

In this study, we investigated whether ubiquitous SRG3 overexpression affects Th2-mediated skin inflammatory diseases such as AD. We found unexpectedly that SRG3^β-actin^ mice develop exacerbated AD. Moreover, we showed that these pathogenic effects are mediated by increased IL4-producing basophils and reduced Foxp3^+^ Treg cells. Therefore, our results demonstrate that modulation of SRG3 function can be applied as one of the options to control AD pathogenesis.

## 2. Results

### 2.1. Ubiquitous SRG3 Overexpression Exacerbates the Pathogenesis of AD in NC Mice

**Figure 1:**
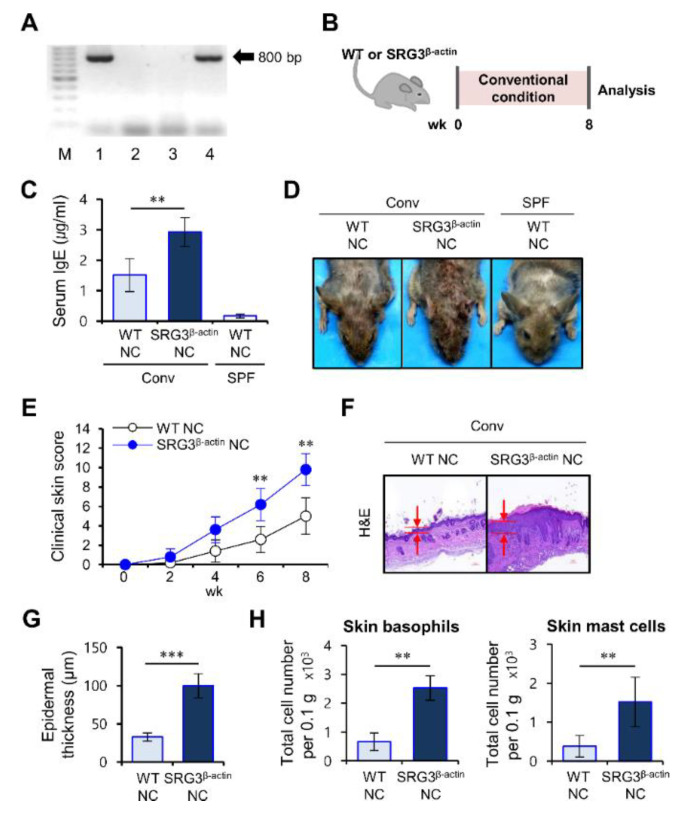
SWI3-related gene (SRG3) overexpression driven by the β-actin promoter exacerbates the severity of atopic dermatitis (AD) development in NC/Nga (NC) mice. (**A**) Genotyping of the SRG3 Tg gene by PCR analysis. SRG3 Tg gene PCR band sizes are 800 bp long. M: marker; Lane 1 (+): positive control (from SRG^β-actin^ Tg B6); Lane 2 (−): negative control (from wild-type (WT) B6); Lane 3: SRG3^β-actin^ non-Tg NC; Lane 4: SRG3^β-actin^ Tg NC. (**B**–**H**) SRG3^β-actin^ NC and WT littermate NC mice maintained under barrier conditions until 6 weeks of age were moved to conventional housing conditions to develop AD spontaneously. WT NC mice housed under normal specific pathogen-free (SPF) conditions are denoted as non-AD conditions. All samples were prepared from mice at 14 weeks of age. Serum IgE levels were measured by ELISA (**C**). The clinical symptoms were measured once a week to monitor the onset of AD (**D**–**E**). The skins were prepared from mice at 14 weeks of age. Skin lesions were sectioned and stained with hematoxylin and eosin (H&E) (**F**). The epidermal thickness was measured in 10 random high-power fields (400×) per sampled lesion (**G**). Mononuclear cells (MNCs) in the skin were isolated from mice shown in Figure 1 (Panel (**A**)). The absolute numbers of mast cells (CD45^+^FcεRI^+^CD200R3^−^CD3^−^CD19^−^) and basophils (CD45^+^FcεRI^+^CD200R3^+^CD3^−^CD19^−^) were determined in the skin by flow cytometry (**H**). The mean values ± SD (*n* = 5; per group in the experiment; Student’s *t*-test; ** *p* < 0.01, *** *p* < 0.001) are shown. One representative experiment of two experiments is shown.

We have previously shown that ubiquitous overexpression of SRG3 protects against EAE pathogenesis via upregulation of Th2 responses [17]. Since the role of SRG3 in skin inflammation is unknown, we explored the impact of ubiquitous SRG3 overexpression on AD development. To address this issue, we developed SRG3^β-actin^ NC mice carrying the β-actin-SRG3 transgene on the AD-susceptible NC genetic background (Figure 1A,B).

Wild-type (WT) and SRG3^β-actin^ NC mice developed skin inflammation spontaneously when raised under conventional housing conditions. After transferring the animals to conventional conditions, these mice were monitored to measure the clinical parameters, including lichenification, edema, erosion/excoriation, scarring/dryness, and erythema/hemorrhage once per week from 6 weeks of age for a total of 8 weeks. Although the severity of AD in WT littermate NC mice gradually but modestly increased by 14 weeks of age, SRG3^β-actin^ NC mice exhibited accelerated disease with exacerbated skin inflammation. Consistent with the enhanced clinical disease scores, SRG3^β-actin^ NC mice displayed increased serum IgE levels and epidermal thickness compared with WT littermate NC mice (Figure 1C–G). Additionally, numbers of skin basophils and mast cells were increased in SRG3^β-actin^ NC mice compared with controls (Figure 1H). Based on our measurement of clinical parameters, including skin inflammation symptoms and serum IgE, we conclude that SRG3 overexpression driven by the β-actin promoter worsens AD’s severity in SRG3^β-actin^ NC mice.

T and natural killer (NK) cell-restricted SRG3 overexpression (driven by the CD2 promoter) exacerbates EAE pathogenesis [17]. We, therefore, asked whether CD2 promoter-driven SRG3 (SRG3^CD2^) overexpression in the NC genetic background modulates AD development. We established SRG3^CD2^ NC mice, which developed attenuated skin inflammation as compared with WT littermate NC mice, in a manner dependent on CD4^+^ T cells (Supplementary Materials Figures S1–S3).

### 2.2. Elevated Levels of M2 Macrophages but Not DCs Correlate with Severe Skin Inflammation in SRG3^β-actin^ NC Mice

Recent studies have reported that the expansion of type 2 antigen-presenting cell (APC) subsets can modulate the development of allergic diseases such as asthma and AD [18,19]. Thus, we considered that the aggravated AD phenotype of SRG3^β-actin^ NC mice might be due to alterations of APCs such as dendritic cells (DCs) and macrophages.

**Figure 2:**
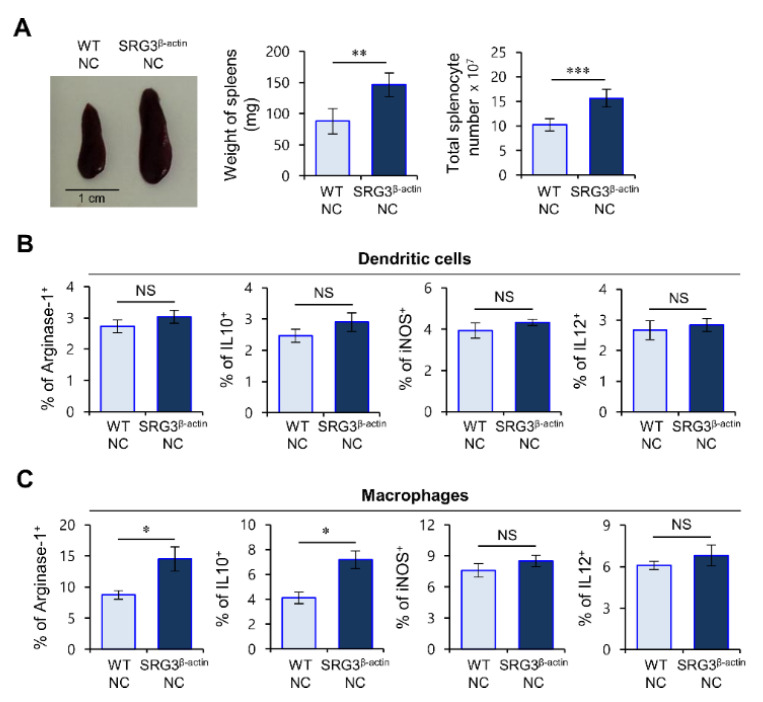
Elevated levels of type 2 phenotype macrophages but not dendritic cells (DCs) correlate with severe skin inflammation in SRG3^β-actin^ mice. (**A**–**C**) SRG3^β-actin^ NC and WT littermate NC mice maintained under barrier conditions were moved to conventional housing conditions at 6 weeks of age to develop AD spontaneously. Splenocytes were prepared from these mice at 14 weeks of age. (**A**) (**Left**), a representative picture of the spleens from AD-induced SRG3^β-actin^ NC and WT littermate NC mice. (**Middle**) and (**Right**), Spleen weight and splenocyte number of these mice. (**B**,**C**) Intracellular arginase-1, interleukin 10 (IL10), inducible nitric oxide synthase (iNOS), and IL12 production were analyzed in DCs (CD11c^+^) (**B**) and macrophages (CD11b^+^F4/80^+^CD11c^−^) (**C**). The mean values ± SD (*n* = 5; per group in the experiment; Student’s *t*-test; * *p* < 0.05, ** *p* < 0.01, *** *p* < 0.001) are shown. One representative experiment of two experiments is shown. NS: no significant.

To address this issue, we analyzed immune response markers on DCs and macrophages (type 1 markers: inducible nitric oxide synthase (iNOS) and interleukin 12 (IL12); type 2 markers: arginase-1 and IL10). DCs and macrophages were isolated from mice with or without AD and were subsequently assessed for expression of type 1 and 2 markers via flow cytometry. SRG3^β-actin^ NC mice without disease displayed a subtle (but not significant) increase in the numbers of DCs and macrophages compared with WT littermate NC mice (Figure S4). Interestingly, upon AD induction, we found that SRG3^β-actin^ NC mice displayed elevated levels of macrophages but not DCs with type 2 phenotypes compared with WT mice (Figure 2A–C). Our results strongly indicate that macrophages rather than DCs are involved in AD pathogenesis due to ubiquitous SRG3 overexpression.

### 2.3. The Effects of SRG3 Overexpression on AD Severity Are Associated with Increased Th2 Responses

**Figure 3:**
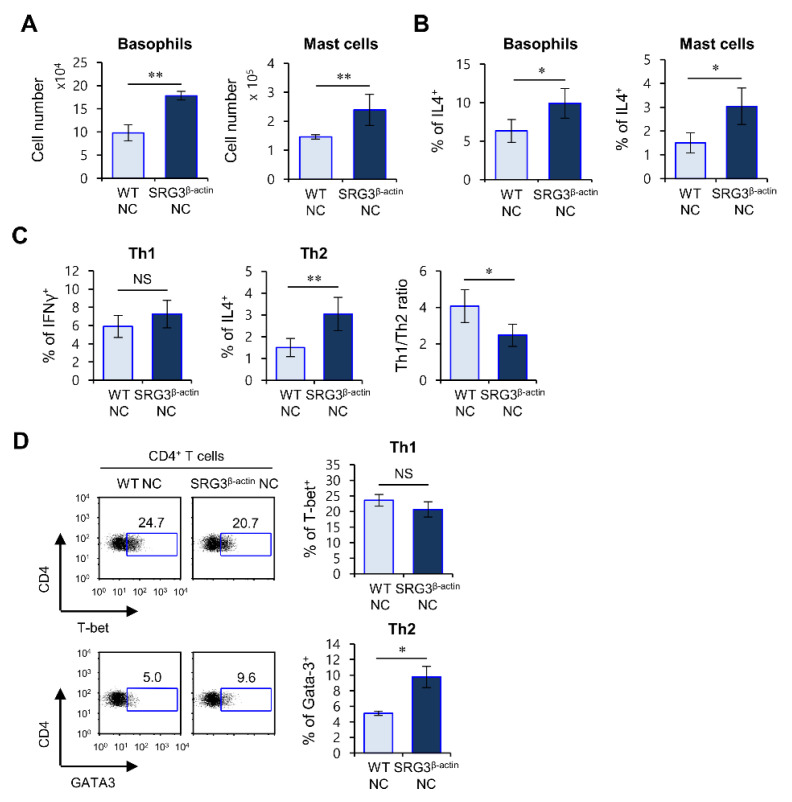
The effects of SRG3 overexpression on the severity of AD are associated with increased Th2 cell responses. (**A**–**D**) SRG3^β-actin^ NC and WT littermate NC mice maintained under barrier conditions were moved to conventional housing conditions at 6 weeks of age to develop AD spontaneously. Splenocytes were prepared from these mice at 14 weeks of age. (**A**) The absolute total cell numbers of both basophils and mast cells were determined in the spleen. (**B**) The percentages of IL4-producing cells among both basophils and mast cells were analyzed via flow cytometry. (**C**) interferon γ (IFNγ)- and IL4-producing populations in splenic CD4^+^ T cells from each group were determined by flow cytometry. (**D**) Intracellular T-bet and GATA binding protein 3 (GATA3) in CD4^+^ T cells were evaluated by flow cytometry. ((**left**) panel) Representative fluorescence-activated cell sorting (FACS) plots. ((**right**) panel) Summary of data. The mean values ± SD (*n* = 5; per group in the experiment; Student’s *t*-test; * *p* < 0.05, ** *p* < 0.01) are shown. One representative experiment of two experiments is shown.

Innate immune cells such as basophils and mast cells can produce IL4, leading to the initiation of Th2 immune responses [3,4]. Therefore, we investigated whether IL4 production and expansion of these innate immune cells correlate with AD pathogenesis in SRG3^β-actin^ NC mice. As expected, SRG3 overexpression significantly elevated the total cell number and IL4 production of basophils and mast cells in the spleen (Figure 3A,B).

Because a previous study showed that elevated basophil numbers and IL4 production affect Th2 polarization, we asked whether β-actin-driven SRG3 overexpression can modulate Th2 cell differentiation during AD development. To test this possibility, we examined Th1/Th2 cytokine profiles of CD4^+^ helper T cells from SRG3^β-actin^ NC mice with fully developed AD via flow cytometric analysis. We found that SRG3^β-actin^ NC mice with AD display a reduction in the Th1/Th2 ratio compared with WT mice (Figure 3C,D). These results indicate that β-actin-mediated SRG3 overexpression upregulates pathogenic Th2 immune responses in NC mice.

### 2.4. β-Actin-Driven SRG3 Overexpression Decreases Treg Cell Differentiation during the Development of AD

It has been reported that Treg cells, critical regulators of inflammation, regulate the development of AD by controlling Th2 cells and serum IgE levels [12]. Moreover, the Th2 cell-derived cytokine IL4 suppresses transforming growth factor β (TGFβ)-mediated polarization of CD4^+^ T cells towards Treg cells [20]. Thus, we wondered whether β-actin-driven SRG3 overexpression can affect the generation of Treg cells in NC mice during AD development.

**Figure 4:**
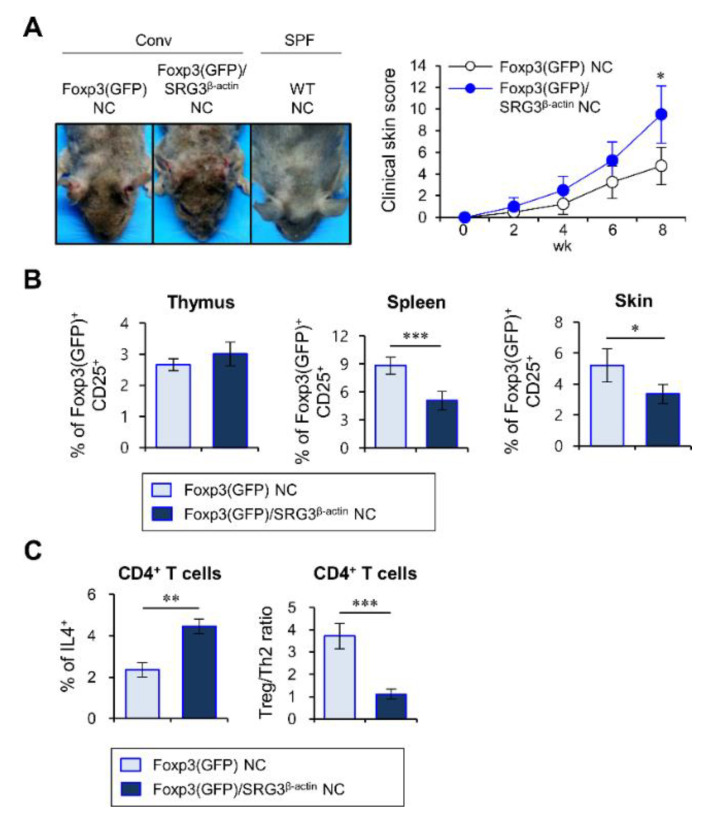
The effects of SRG3 overexpression on the severity of AD are associated with a reduction in the frequency of Treg cells. (**A**–**C**) Foxp3(GFP)/SRG3^β-actin^ NC and Foxp3(GFP) littermate NC mice maintained under barrier conditions were moved to conventional housing conditions at 6 weeks of age. All the samples were prepared from mice at 14 weeks of age. (**A**) The clinical symptoms were measured once a week to monitor the onset of AD. (**B**) Thymocytes, splenocytes, and skin-derived mononuclear cells were prepared from the two groups. The percentage of Foxp3(GFP)^+^CD25^+^ cells among CD4^+^ T cells from the thymus, spleen, and skin of each group was evaluated by flow cytometry. (**C**) The percentage of IL4^+^ cells and the ratio of Foxp3(GFP)^+^ cells/IL4^+^ cells among CD4^+^ T cells from each group’s spleen were evaluated by flow cytometry. The mean values ± SD (*n* = 4; per group in the experiment; Student’s *t*-test; * *p* < 0.05, ** *p* < 0.01, *** *p* < 0.001) are shown.

To address this issue, we generated Foxp3(GFP)/SRG3^β-actin^ NC mice by crossing Foxp3(GFP) reporter NC mice with SRG3^β-actin^ NC mice and subsequently evaluated the spontaneous AD development and accompanying clinical symptoms. We found that Foxp3(GFP)/SRG3^β-actin^ NC mice developed AD more severely and rapidly than the control Foxp3(GFP) littermate NC mice (Figure 4A). Next, to examine whether this was associated with numerical changes of Treg cells, we measured the frequency of Treg cells in various organs, including the thymus, spleen, and skin. Compared to Foxp3(GFP) littermate NC mice, we found no significant difference in the frequency of thymic Treg cells in Foxp3(GFP)/SRG3^β-actin^ NC mice. However, we found that the frequencies of Treg cells in both the spleen and the skin were dramatically decreased in Foxp3(GFP)/SRG3^β-actin^ NC mice compared to Foxp3(GFP) littermate NC mice (Figure 4B). Furthermore, upon AD induction, Foxp3(GFP)/SRG3^β-actin^ NC mice showed a reduction of the Treg/Th2 cell ratio compared with Foxp3(GFP) littermate NC mice (Figure 4C). Overall, these results indicate that alterations of Treg cells due to β-actin-mediated SRG3 overexpression might be associated with the severity of AD pathogenesis.

## 3. Discussion

In this study, we show that SRG3^β-actin^ NC mice are enriched for M2-type macrophages compared to WT littermate NC mice, a finding consistent with our prior report that β-actin promoter-driven SRG3 overexpression in B6 mice polarizes macrophages towards an arginase-1-expressing M2 phenotype during EAE [17]. The differentiation of M2 macrophages is critically dependent on the cytokine IL4. Since basophil-derived IL4 can promote M2 macrophages [21], a dramatic increase of IL4^+^ basophils and mast cells may account for the increased M2 phenotype observed in SRG3^β-actin^ NC mice. In addition to IL4-producing cells, Treg cells have a crucial role in controlling AD in mouse models and human subjects [12,13]. Furthermore, previous studies have shown that Th2 cell-derived IL4 inhibits the differentiation and suppressive function of Treg cells [20,22]. We found that Foxp3(GFP)^+^CD25^+^ Treg cells are dramatically reduced in the spleen and skin of SRG3^β-actin^ NC mice, implying the suppression of Treg cells by increased IL4^+^ immune cells. Thus, expansion of IL4-producing immune cells (e.g., basophils, mast cells, and Th2 cells) and consequent reduction of Treg cells can induce type 2-dominant immunity that enhances allergic immune responses in SRG3^β-actin^ NC mice, consequently resulting in aggravated AD.

Although AD pathogenesis is related to impaired Th1/Th2 balance by an increased Th2-type immune response, it has been reported that Th17-type immune responses also contribute to exacerbating different types of AD [23]. Not only IL6 but also IL23 is essential in the pathogenesis of Th17-type AD [24]. Thus, it will be worthwhile to investigate whether the enhancement of SRG3 expression affects the production of Th17-type cytokines (IL6 and IL23) by DCs and macrophages during AD development.

Furthermore, it has been reported that a significant decrease in the skin’s filaggrin expression correlates with a dysfunctional skin barrier in AD [25]. Moreover, skin cells (epidermal keratinocytes and dermal fibroblasts) participate in initiating skin inflammation in AD. In particular, the production of pro-Th2 cytokines (thymic stromal lymphopoietin and IL33) by keratinocytes and fibroblasts plays a critical role in the induction stage of skin inflammation of AD [26,27]. In this regard, it will be interesting to investigate further the effects of SRG3 overexpression on filaggrin expression and keratinocyte/fibroblast functions via cell-type-specific gene modification.

Based on our findings, lymphocytes (particularly CD4^+^ T cells) from SRG3^CD2^ NC mice are responsible for attenuating the development of AD. In contrast, non-lymphocyte populations (particularly M2 macrophages) from SRG3^β-actin^ NC mice can override the protective SRG3-overexpressing CD4^+^ T cells during AD development. One recent work demonstrated that microRNAs such as miR-221 and miR-222 negatively regulate functions of BRG1, a component of the SWI/SNF complex by binding to specific sites within the 3′ UTR region of BRG1 mRNA, by which microRNAs indirectly downregulate BRG1-associated inflammatory gene expression in macrophages [28]. This study implies that the activities of chromatin remodeling factors including SWI/SNF complex may be regulated in a more intricate manner.

However, it is still difficult to exclude the possibility that non-immune cells, including endothelial cells, keratinocytes, and fibroblasts, play essential roles in AD development from the standpoint of SRG3 overexpression. Moreover, one recent study demonstrated that BAF155, the human homolog of SRG3, is highly expressed in nasal epithelial cells of chronic rhinosinusitis (CRS) patients [29]. Its expression significantly correlates with allergy symptoms in CRS patients. This study suggests the clinical importance of SRG3 expression in non-immune cells such as epithelial cells. Thus, more efforts should be made to identify the exact cell types involved in the pathogenesis of AD development and to reveal the cellular and molecular mechanism of AD pathogenesis.

Our findings suggest the following scenario for the capacity of β-actin promoter-driven SRG3 overexpression to exaggerate AD development. First, SRG3 overexpression somehow expands basophils and mast cells and also induces them to produce IL4. Second, increased IL4 in SRG3^β-actin^ NC mice polarizes macrophages toward an M2 phenotype, promotes Th2 cell differentiation, and possibly suppresses Treg cell differentiation. Finally, SRG3 overexpression-induced imbalance between Treg cells and Th2 cells causes inappropriate allergic responses, resulting in uncontrolled AD development. Therefore, our results provide evidence that SRG3 might be a therapeutic target to modulate inflammatory skin diseases such as AD. Our recent study showed that genetic engineering of PD-L1 and PD-L2 on cancer cells via CRISPR/Cas9 approaches can improve IFNγ secretion by CD8^+^ T cells [30]. The expression of PD-L1 and PD-L2 in dermal fibroblasts is upregulated by activated T cells in the skin of the alopecia areata mouse model, suggesting that gene modification of the PD-1/PD-L1 checkpoint pathway provides an immunotherapeutic strategy against skin immune diseases [31]. Thus, it will be interesting to examine whether combined therapy using genetic engineering of both SRG3 and PD-L1/PD-L2 expression can effectively control inflammatory skin disease pathogenesis.

## 4. Materials and Methods

### 4.1. Mice and Reagents

WT B6 mice were purchased from Jung Ang Lab Animal Inc. (Seoul, Korea). SRG3^β-actin^ B6, SRG3^CD2^ B6, and Foxp3(GFP) reporter B6 mice were provided by Dr. Rho H. Seong (Seoul National University, Seoul, Korea). SRG3^β-actin^ B6, SRG3^CD2^ B6, and Foxp3(GFP) reporter B6 mice were backcrossed to NC mice for more than eleven generations. Foxp3(GFP) reporter NC mice were further crossed with SRG3^β-actin^ NC mice to obtain Foxp3(GFP)/SRG3^β-actin^ NC mice. These mice were maintained at Sejong University and were used for experiments at 6–12 weeks of age. They were maintained on a 12-h light/12-h dark cycle in a temperature-controlled barrier facility with free access to food and water. Mice were fed a γ-irradiated sterile diet and provided with autoclaved tap water. For monitoring spontaneous AD development, mice were transferred at 6 weeks of age from the barrier facility to the conventional animal facility. Age- and sex-matched mice were used for all experiments. The animal experiments were approved by the Institutional Animal Care and Use Committee of Sejong University (SJ-20161104).

### 4.2. Genotyping of Mice

To verify the transgene’s integration, genomic DNA from tail biopsies was used to amplify an 800 bp fragment that was only detectable in SRG3^β-actin^ mice carrying the SRG3 transgene. The following primers were used for genotyping SRG3^β-actin^ mice by PCR: forward 5′-GAC TAG ACC AAA CAT CTA CCT C-3′; and reverse 5′- GTC AAC TGA GCG ACT GGA TC-3′. This process is consistent with the protocol used in our previous study [17].

### 4.3. Cell Isolation and Culture

Splenic CD4^+^ T cells were isolated from NC mice using a magnetically activated cell sorting (MACS) system (Miltenyi Biotec, Bergisch Gladbach, Germany), following the manufacturer’s instructions. CD4^+^ T cells were enriched >96% after MACS. The purity of the enriched cells was more than 97%. Primary cells were cultured in RPMI 1640 (Gibco BRL, Grand Island, NY, USA) culture media supplemented with 10% fetal bovine serum (FBS), 10 mM hydroxyethyl piperazine ethane sulfonic acid (HEPES), 2 mM L-glutamine, 100 units/mL penicillin-streptomycin, and 5 μM 2-mercaptoethanol.

### 4.4. Flow Cytometry

The following monoclonal antibodies (mAbs) were obtained from BD Biosciences (San Jose, CA, USA): fluorescein isothiocyanate (FITC)-, phycoerythrin (PE)-Cy7- or allophycocyanin (APC)-conjugated anti-CD3ε (clone 145-2C11); FITC-, PE-Cy7, or APC-conjugated anti-CD4 (clone RM4-5); FITC- or APC-conjugated anti-CD45 (clone 30-F11); APC-conjugated anti-CD25 (clone PC61); PE-conjugated anti-T-bet (clone 4B10); PE-conjugated anti-GATA3 (clone L50-823); PE-Cy7-conjugated anti-CD11b (clone M1/70); FITC- or APC-conjugated anti-CD11c (clone HL3); PE-conjugated anti-IL10 (clone JES5-16E3); PE-conjugated anti-IL12p40 (clone C15.6); PE-conjugated anti-IgG1 (κ isotype control). The following mAbs were purchased from eBioscience: APC-conjugated anti-CD200R3 (clone Ba13); FITC- or APC-conjugated anti-CD19 (clone ID3); FITC- or PE-conjugated anti-IL4 (clone BVD6-24G2); APC-conjugated anti-F4/80 (clone BM8); PE-conjugated anti-iNOS (clone CXNFT); FITC- or APC-conjugated anti-FcεRI (clone MAR-1); PE-conjugated anti-IFNγ (clone XMG1.2). The following mAb from R&D Systems (Minneapolis, MN, USA) was used: PE-conjugated anti-Arginase-1. For staining surface markers, cells were harvested and washed twice with cold 0.5% BSA-containing PBS (FACS buffer). For blocking Fc receptors, the cells were incubated with anti-CD16/CD32 mAbs on ice for 10 min and subsequently stained with fluorescently-labeled mAbs. Flow cytometric data were acquired using a FACSCalibur flow cytometer (Becton Dickson, San Jose, CA, USA) and analyzed using FlowJo software (Tree Star Inc., Ashland, OR, USA).

### 4.5. Intracellular Cytokine Staining

For intracellular staining, splenocytes were incubated with brefeldin A, an intracellular protein transport inhibitor (10 μg/mL), in RPMI medium for 2 h at 37 °C. The cells were stained for cell surface markers, fixed with 1% paraformaldehyde, washed once with cold FACS buffer, and permeabilized with 0.5% saponin. The permeabilized cells were then stained for an additional 30 min at room temperature with the indicated mAbs (PE-conjugated anti-IFNγ, anti-IL4, anti-IL12, anti-IL10, anti-iNOS, anti-Arginase1, or PE-conjugated isotype control rat IgG mAbs). Fixation and permeabilization were performed using a Foxp3 staining kit (eBioscience, San Diego, CA, USA) with the indicated mAbs (PE-conjugated anti-T-bet, anti-GATA3, or isotype control rat IgG mAbs). More than 5000 cells per sample were acquired using a FACSCalibur, and the data were analyzed using the FlowJo software package (Tree Star, Ashland, OR, USA).

### 4.6. Preparation of Skin Cell Suspensions

The skin was dissected and dermal fat was removed with scissors. The tissue was cut into small pieces with a scalpel and digested with 2.5 mg/mL collagenase type IV (Sigma, St. Louis, MO, USA) and 1 mg/mL DNase I (Promega, Madison, WI, USA) for 4 h at 37 °C. At the end of the incubation, the digested tissue was dissociated into single-cell suspensions using gentleMACS Dissociator (Miltenyi, Germany) in combination with C Tubes. Single-cell suspensions were smashed through a 70 μm nylon cell strainer (BD Falcon, Franklin Lakes, NJ, USA) and collected in a 50 mL Falcon tube. The cells were washed once with PBS + 10% FBS (1400 rpm, 10 min, 4 °C), and subsequently separated with 37%/70% Percoll (GE Healthcare, Chicago, IL, USA) gradients. Mononuclear cells were collected from the layer that was below 37% and above 70% gradient. After washing with PBS, the total mononuclear cell number was determined using a hemacytometer with 0.4% trypan blue (Welgene, Gyeongsan-si, Korea) before antibody staining.

### 4.7. ELISA

Serum IgE levels were measured with a sandwich ELISA (clone R35-72 for capturing IgE and R35-118 for detecting IgE; BD PharMingen, San Jose, CA, USA). The optical density was measured at 450 nm with an immunoreader (Bio-Tek ELX-800, Winooski, VT, USA).

### 4.8. Analysis of Skin Sections

Dorsal skin was fixed in 4% paraformaldehyde, embedded in paraffin, and cut into six μm sections using a microtome (RM 2235, Leica, Wetzlar, Germany). The sections were then stained with hematoxylin and eosin (H&E) to analyze histological changes and stained with toluidine blue to detect mast cells. The cells were counted with a microscope at a magnification of 400 times. The cell density was expressed as the total number of cells in ten high-power fields (400×) for each section.

### 4.9. Scoring the Severity of Skin Lesions

Skin lesions were scored at the indicated time points. The scoring was based on the severity of lichenification, edema, erosion/excoriation, scarring/dryness, and erythema/hemorrhage. The total clinical skin severity score was defined as the sum of the five signs (none = 0; mild = 1; moderate = 2; and severe = 3). SRG3^β-actin^ and SRG3^CD2^ NC mice were moved to conventional housing conditions at six weeks of age to develop AD spontaneously. The clinical symptoms of SRG3^β-actin^ and SRG3^CD2^ NC mice were measured once a week from 6 weeks of age for a total of 8 and 12 weeks, respectively.

### 4.10. Statistical Analysis

Statistical significance was determined using Excel (Microsoft, Redmond, WA, USA). Student’s *t*-test was performed for the comparison of two groups. * *p* < 0.05, ** *p* < 0.01, and *** *p* < 0.001 were considered significant in the Student’s *t*-test.

## 5. Conclusions

In summary, our results demonstrate that β-actin promoter-driven SRG3 overexpression exacerbates AD symptoms in NC mice. The significant increase of Th2 cell differentiation in SRG3^β-actin^ NC mice was attributable to the SRG3 overexpression-mediated induction of AD pathogenesis. Moreover, these effects are closely related to increased IL4-producing basophils and reduced Foxp3^+^ Treg cells. Therefore, our results provide evidence that ubiquitous SRG3 overexpression exhibits pathogenic effects on AD development in NC mice.

## Data Availability

The data will be available from the corresponding author on reasonable request.

## Supplementary Materials

The following are available online at https://www.mdpi.com/1422-0067/22/4/1553/s1.
