# Supplementary material for: Ubiquitous Overexpression of Chromatin Remodeling Factor SRG3 Exacerbates Atopic Dermatitis in NC/Nga Mice by Enhancing Th2 Immune Responses
Source: Int J Mol Sci. 2021 Feb 4;22(4):1553. doi: 10.3390/ijms22041553 (PMC7913833; doi:10.3390/ijms22041553)
Supplement: Supplementary file 1 [file ijms-22-01553-s001.pdf]

**Supplementary Materials to:**

# Ubiquitous Overexpression of Chromatin Remodeling Factor SRG3 Exacerbates Atopic Dermatitis in NC/Nga mice by Enhancing Th2 Immune Responses

**This document includes:**

**-Supplementary figures 1-4**

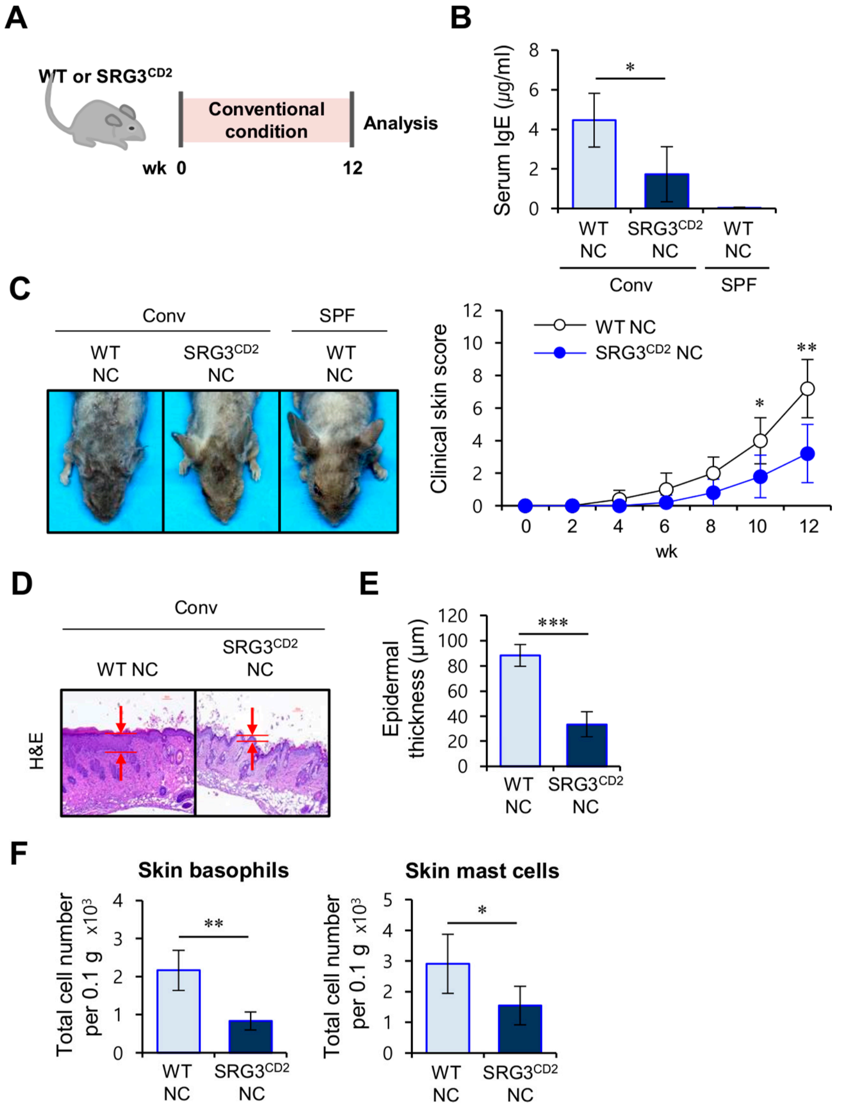


**FIGURE S1. CD2 promoter-driven SRG3 overexpression protects NC mice from AD development.**

SRG3$^{CD2}$ NC and WT littermate NC mice maintained under barrier conditions were moved to conventional housing conditions at 6 weeks of age to develop AD spontaneously. All the samples were prepared from these mice at the age of 18 weeks. (A) Experimental scheme. (B) Serum IgE levels from the indicated mice were measured by ELISA. (C) The clinical symptoms were measured once a week to monitor the onset of AD. (D-F) Skins were prepared from SRG3$^{CD2}$ NC and WT littermate NC mice. (D) Skin lesions were sectioned and stained with H&E. (E) The epidermal thickness was measured in 10 random high-power fields (400×) per sampled lesion. (F) MNCs in the skin were isolated from WT littermate NC and SRG3$^{CD2}$ NC mice. The absolute cell numbers

of mast cells ($CD45^{+}Fc\varepsilon RI^{+}CD200R3^{-}CD3^{-}CD19^{-}$) and basophils ($CD45^{+}Fc\varepsilon RI^{+}CD200R3^{+}CD3^{-}CD19^{-}$) in the skin were determined by flow cytometry. The mean values ± SD (*n* = 5; per group in the experiment; Student's t-test; *P<0.05, **P<0.01, ***P<0.001) are shown. One representative experiment of two experiments is shown.

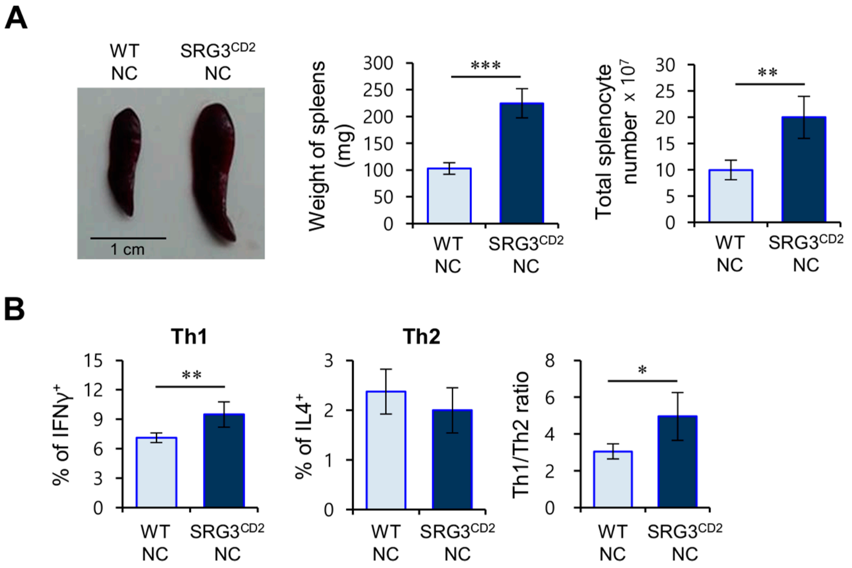


**FIGURE S2. The AD protective effects of CD2 promoter-driven SRG3 overexpression are associated with increased Th1 responses.**

(A-B) Spleens were prepared from WT littermate NC and SRG3$^{CD2}$ NC mice. (A) Left, a representative picture of the spleens from AD-induced WT littermate NC and SRG3$^{CD2}$ NC mice. Middle and Right, Spleen weight and splenocyte number of these mice. (B) The frequencies of IFNγ- and IL4-producing populations in splenic CD4$^{+}$ T cells from each group were determined by flow cytometry. The mean values ± SD (*n* = 5; per group in the experiment; Student's t-test; *P<0.05, **P<0.01, ***P<0.001) are shown. One representative experiment of two experiments is shown.

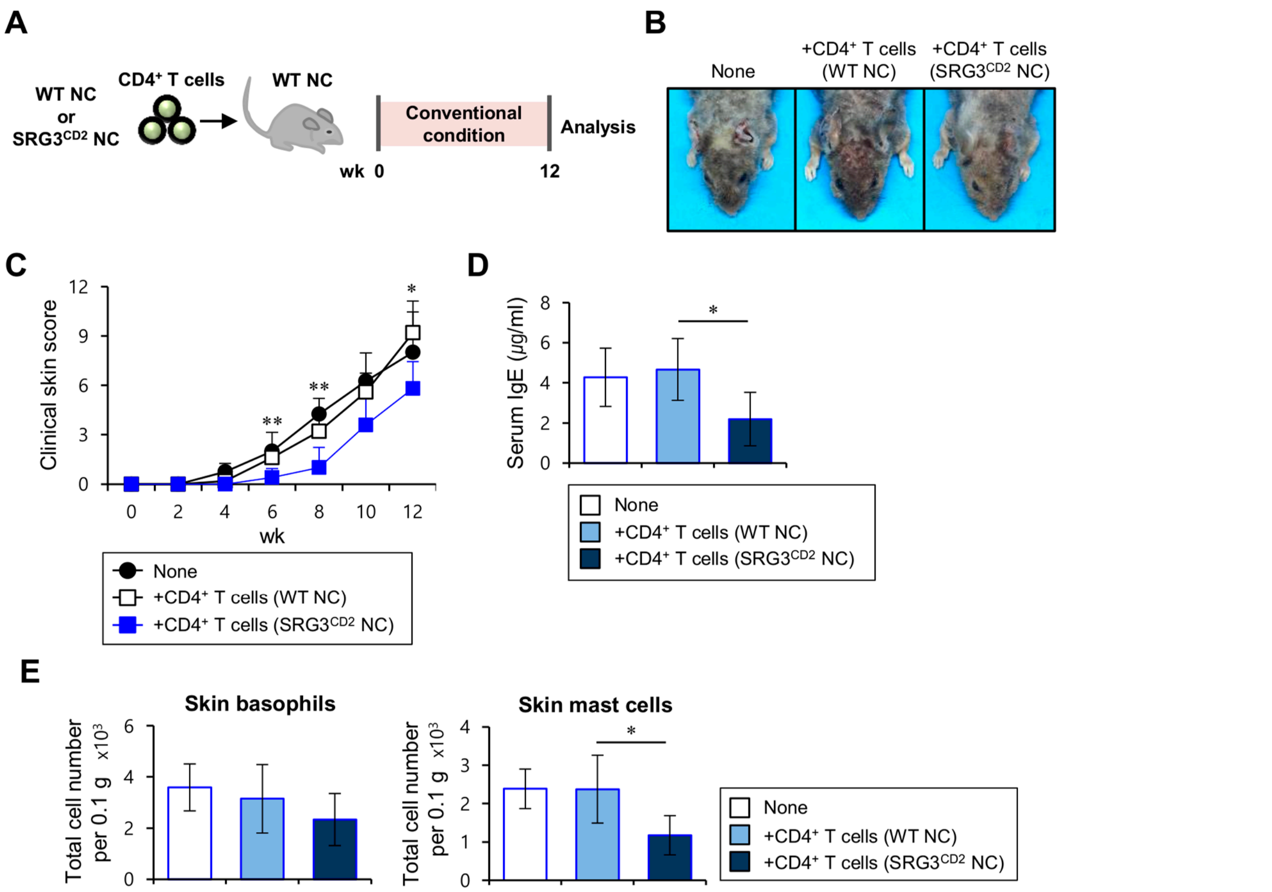


**FIGURE S3. Adoptive transfer of CD4$^+$ T cells from SRG3$^{CD2}$ NC mice but not WT NC mice protects recipient NC mice from AD development.**

(A-E) Splenic CD4$^+$ T cells were purified from SRG3$^{CD2}$ NC and WT littermate NC mice and subsequently stimulated with PMA/Ionomycin for 4 hrs. Activated CD4$^+$ T cells ($5 \times 10^6$) from these mice were adoptively transferred into WT NC mice, which were then maintained under conventional housing conditions to develop AD for 12 weeks spontaneously. (B-C) The clinical symptoms were measured once a week to monitor the onset of AD. (D) Serum IgE levels were measured by ELISA. (E) MNCs in the skin were isolated from SRG3$^{CD2}$ NC and WT littermate NC mice. The absolute cell numbers of mast cells (CD45$^+$FcεRI$^+$CD200R3$^-$CD3$^-$CD19$^-$) and basophils (CD45$^+$FcεRI$^+$CD200R3$^+$CD3$^-$CD19$^-$) in the skin were determined by flow cytometry. The mean values ± SD ($n$ = 5; per group in the experiment; Student's t-test; *$P<0.05$) are shown.

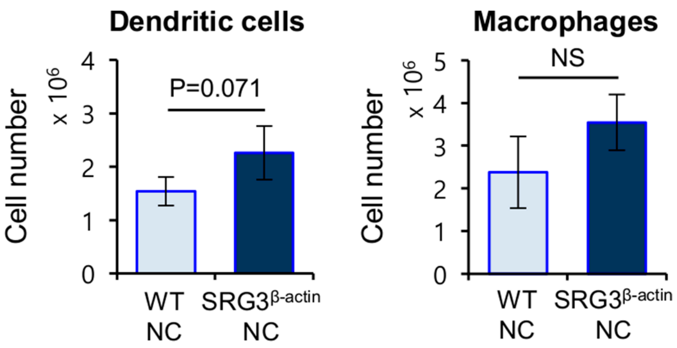


**Figure S4. Effects of SRG3 overexpression on the cellularity of DCs and macrophages in NC mice with AD.**

The spleens were isolated from SRG3$^{β\text{-actin}}$ NC and WT littermate NC mice. The absolute cell numbers of splenic DCs and macrophages were determined by flow cytometry. The mean values ± SD ($n$ = 4; per group in the experiment) are shown. One representative experiment of two experiments is shown.
